# Examining conscientiousness as a key resource in resisting email interruptions: Implications for volatile resources and goal achievement

**DOI:** 10.1111/joop.12177

**Published:** 2017-05-18

**Authors:** Emma Russell, Stephen A. Woods, Adrian P. Banks

**Affiliations:** ^1^ WWK Research Group Kingston Business School Kingston University Surrey UK; ^2^ Department of People and Organisations University of Surrey Guildford UK; ^3^ Department of Psychology University of Surrey Guildford UK

**Keywords:** conscientiousness, conservation of resources, email, interruptions, resources, self‐control, trait activation

## Abstract

Within the context of the conservation of resources model, when a resource is deployed, it is depleted – albeit temporarily. However, when a ‘key’, stable resource, such as Conscientiousness, is activated (e.g., using a self‐control strategy, such as resisting an email interruption), we predicted that (1) another, more volatile resource (affective well‐being) would be impacted and that (2) this strategy would be deployed as a trade‐off, allowing one to satisfy task goals, at the expense of well‐being goals. We conducted an experience‐sampling field study with 52 email‐users dealing with their normal email as it interrupted them over the course of a half‐day period. This amounted to a total of 376 email reported across the sample. Results were analysed using random coefficient hierarchical linear modelling and included cross‐level interactions for Conscientiousness with strategy and well‐being. Our first prediction was supported – deploying the stable, key resource of Conscientiousness depletes the volatile, fluctuating resource of affective well‐being. However, our second prediction was not fully realized. Although resisting or avoiding an email interruption was perceived to hinder well‐being goal achievement by Conscientious people, it had neither a positive nor negative impact on task goal achievement. Implications for theory and practice are discussed.

**Practitioner points:**

- It may be necessary for highly Conscientious people to turn off their email interruption alerts at work, in order to avoid the strain that results from an activation‐resistance mechanism afforded by the arrival of a new email.
- Deploying key resources means that volatile resources may be differentially spent, depending on one's natural tendencies and how these interact with the work task and context. This suggests that the relationship between demands and resources is not always direct and predictable.
- Practitioners may wish to appraise the strategies they use to deal with demands such as email at work, to identify if these strategies are assisting with task or well‐being goal achievement, or whether they have become defunct through automation.

## Background

Within the context of the conservation of resources (COR) model, there has been much debate about what constitutes a ‘resource’. Hobfoll (1988, 1989) conceptualizes a resource as anything which one values, or which protects and builds something of value (Hobfoll, 2001). Halbesleben, Neveu, Paustian‐Underdahl, and Westman (2014) extend this definition by asserting that a resource is, ‘anything perceived by the individual to help attain his or her goals’ (p. 5), inferring that what is considered to be a resource will depend upon which goals are being pursued at any time. Recent research in the field of personality indicates that traits orient us towards our goals, allowing us to garner meaning and a sense of purpose when such goals are realized (Barrick, Mount, & Li, 2013). This supposes that traits are likely to be involved in the deployment of resources to achieve trait‐relevant goals.

Traits are also seen as resources in their own right (Judge & Zapata, 2015). For example, in COR, Conscientiousness is considered to be a ‘key’ resource (Gorgievski, Halbesleben, & Bakker, 2011) used to oversee the application of lower order, less stable resources (Hobfoll, 2011) during goal pursuit (Halbesleben *et al*., 2014). Lower‐order resources (e.g., time and energy) are more volatile and expendable (ten Brummelhuis & Bakker, 2012), whereas key resources are less readily spent as they tend to be stable, durable and not easily transferred (ten Brummelhuis & Bakker, 2012). Yet, central to Hobfoll's original COR model is the notion that in using resources, we deplete them, and this resource loss can result in feelings of strain (Halbesleben & Buckley, 2004; Hobfoll, 2002). According to Trait Activation Theory (TAT: Tett & Burnett, 2003), however, activating a personality trait will strengthen rather than deplete it. This poses the question: if using a key resource – such as Conscientiousness – does not deplete that same resource, what instead is impacted?

This study examines Conscientiousness as a key resource and trait, and explores its deployment in response to the presence of a commonly occurring workplace temptation – the email interruption. Email interruptions are a pervasive occurrence in modern work that can disrupt and alter our goal‐directed behaviour (Altmann & Trafton, 2002; Czerwinski, Horvitz, & Wilhite, 2004; Speier, Vessey, & Valacich, 2003). Yet simultaneously, email potentially presents new, important work tasks (Brown, Duck, & Jimmieson, 2014; Dabbish & Kraut, 2006) and is considered to be a work critical tool (Mano & Mesch, 2010; Renaud, Ramsay, & Hair, 2006; Sumecki, Chipulu, & Ojiako, 2011). Throughout this paper, we argue that being presented with an email interruption at work represents a highly salient set of contextual cues for people high on Conscientiousness. On the one hand, the arrival of the email represents a *temptation* in the sense that workers are more likely to feel a desire or duty to check and respond. However, we propose that the context also serves to activate trait‐relevant self‐control behavioural strategies for those higher in Conscientiousness, because ‘resisting’ the email interruption, and rather focusing on the task at hand, demonstrates perseverance and self‐discipline, which are central to the construct of Conscientiousness (Barrick & Mount, 1991).

In resisting email interruptions, Conscientious people must exert self‐control behavioural strategies to achieve their current task goals, but this may come ‘at a price’. For example, exerting self‐control has been shown to be draining and can have a negative impact on well‐being goals (Hobfoll, Johnson, Ennis, & Jackson, 2003; Lin, Ma, Wang, & Wang, 2015). This indicates that activating a trait‐relevant ‘key’ resource may have a depleting effect on volatile resources, such as affective well‐being, and may also have both positive and negative implications for goals. However, the literature on COR theory to date does not fully explain this potentially important mechanism, and neither are we aware of any data available upon which to base the explication, in order to better understand the repercussions of resource activity at work. We address this notable gap in this study.

Our aims in this study are twofold. Firstly, we wish to elucidate an under‐developed concept in COR by specifying whether deploying a key, stable resource will result in the depletion of another, more volatile resource. COR explicitly outlines a resources dimension whereby some resources (e.g., affective well‐being) are at the ‘volatile’ end of the spectrum, readily used and spent, whereas the other, stable end of the spectrum consists of key resources that are more durable, such as personality traits (ten Brummelhuis & Bakker, 2012; Gorgievski *et al*., 2011). COR research frequently returns reports about how resources from one domain or category may build resources in another domain or category (e.g., through resource spirals: Hobfoll, 2002; Perry, Witt, Penney, & Atwater, 2010). Yet, depletion of resources is only discussed in terms of demand or stressor exposure, rather than alternative resource use. In this study, we wish to understand whether applying a resource from the stable end of the spectrum (i.e., Conscientiousness) could deplete resources at the volatile end (i.e., affective well‐being: AWB). AWB is defined as an important component of psychological well‐being that represents hedonic tone and activation of an emotional state at any designated point in time (Daniels, 2000; van Horn, Taris, Schaufeli, & Schreurs, 2004; Warr, 1990). As a temporal construct, AWB fulfils the definition of a volatile resource in COR terms (ten Brummelhuis & Bakker, 2012; Hagger, Wood, Stiff, & Chatzisarantis, 2010).

Secondly, we aim to examine why Conscientious people might deploy self‐control strategies for dealing with email interruptions, if these have a negative impact on their AWB. TAT argues that when traits are activated we feel more satisfied (Tett & Burnett, 2003). However, using Hockey's (1997, 2000, 2002) compensatory control model, we suggest that resource deployment is associated with a trade‐off between satisfying task or well‐being goals, with Conscientious people more disposed to prioritize task goals in resource deployment because of their hard‐working, achievement focused nature (Lin *et al*., 2015; Tangney, Baumeister, & Boone, 2004). This firmly places goal orientation to the centre of resource theory (Halbesleben *et al*., 2014) and indicates that in order to understand how resources are likely to be used in the face of demands at work it is imperative for us to understand towards which goals people are oriented. TAT indicates that goal orientation is strongly associated with core traits, allowing us to make predictions about how and why different people deploy their resources towards different goals at work (Barrick *et al*., 2013).

In the following sections, we first discuss the concept of Conscientiousness as a resource that confers superior resource‐building strategies pertaining to the individual's goals. Next, we outline the definition of an email interruption in this context, emphasizing its emergence as a significant temptation that requires strategic management in modern work environments. Self‐control, as a specific resource deployment strategy of Conscientiousness, is examined in relation to the research associated with self‐control exertion when faced with temptations at work. Finally, we present our hypotheses and test these using an experience‐sampling design with email workers operating in their ‘real’ working environments.

### Conscientiousness as a trait and a resource

We conceptualize personality as, ‘…dimensions of individual differences in tendencies to show consistent patterns of thoughts, feelings and actions’ (Costa & McCrae, 1997, p. 270). As a stable personality trait, Conscientiousness is defined as being organized, hard‐working, careful, and possessing self‐control (George, Helson, & John, 2011). Conscientiousness is clearly conceptualized as a resource in Hobfoll's COR Theory (1989, 2002, 2011), and, according to Halbesleben *et al*.'s (2014) and Halbesleben, Harvey, and Bolino (2009) definitions; it is something that is valued and enables people to acquire other valued resources in the pursuit of salient goals. The positive benefits of Conscientiousness as a resource that confers goal‐related advantages are, for example, underlined in research showing its associations with better work performance (Barrick, Stewart, & Piotrowski, 2002; Witt, Burke, Barrick, & Mount, 2002), and an ability to gather more of – and make better use of – other resources (ten Brummelhuis & Bakker, 2012; Perry *et al*., 2010).

Among the behavioural strategies associated with high Conscientiousness are self‐discipline, perseverance, and self‐control (Costa & McCrae, 1997), which collectively overrule one's natural tendencies to think, feel, or behave in particular ways (Muraven & Baumeister, 2000). For example, when faced with temptations, applying self‐control will override our natural urges to succumb to the temptation. However, empirical studies show that repeated exertion of self‐control is draining (Fishbach, Friedman, & Kruglanski, 2003; Frei, Racio, & Travagline, 1999), and becomes increasingly difficult as energy resources are depleted (Baumeister, Vohs, & Tice, 2007; Muraven & Baumeister, 2000; Vohs & Schmeichel, 2003; Vohs, Baumeister, & Schmeichel, 2012). Such findings appear to stand in contradiction to TAT, which rather proposes that trait‐relevant behaviours (like self‐control in the case of Conscientiousness), are strengthened and deepened by repeated activation (Tett & Burnett, 2003).

However, we propose that these observations may be reconciled by considering the interplay of resources and their effects on one another. If self‐control is conceptualized as a strategy that draws on two resources, one trait‐like (e.g., Conscientiousness) that is stable, and another state‐like (e.g., affect/energy) that is volatile, it is possible to reason that the impact of deployment of the respective resources may result in differentiated effects on each. Conscientiousness is most likely to remain stable or be strengthened, as proposed by TAT, whereas affective resources may by contrast be plundered (Fishbach *et al*., 2003; Frei *et al*., 1999). In effect, the expression or execution of Conscientiousness in behavioural strategies could result in a trade‐off represented in the substantial depletion of other resources. Understanding this interplay could add clarification to our understanding of how resources are utilized in COR (Halbesleben *et al*., 2014). Moreover, understanding how different resources are deployed, spent, and valued is imperative if work psychologists and job designers wish to promote work environments that allow individuals to work to their optimal potential, and deal with daily demands in effective and productive ways (Gorgievski *et al*., 2011).

### Conscientiousness and email interruptions

One of the most ubiquitous ‘temptations’ in modern office life is that of the email interruption. Whilst people have different strategies for dealing with email, research generally indicates that when alerted to the arrival of a new email, people's most common response is to look at it straight away (Jackson, Dawson, & Wilson, 2001; Thomas *et al*., 2006). However, this might be an inefficient strategy as it interferes with performance on the current task (Czerwinski *et al*., 2004), has a negative impact on load and memory processes (Altmann & Trafton, 2002; Einstein, McDaniel, Williford, Pagan, & Dismukes, 2003; Morgan, Patrick, Waldron, King, & Patrick, 2009; Brumby, Cox, Back, & Gould, 2013), and can result in stress or reduced feelings of well‐being (Bailey, Konstan, & Carlis, 2001; Brown *et al*., 2014; Mazmanian, Orlikowski, & Yates, 2005).

We acknowledge that email can be received in a number of different formats. For the purpose of this study, we conceptualize an email interruption as something that unexpectedly alerts us to its presence during the execution of another task, and which affords a task in its own right (van den Berg, Roe, Zijlstra, & Krediet, 1996; Speier *et al*., 2003). To be interrupted by an email then, one must be connected to the email system whilst undertaking other work, with alerts (audible and/or visual) switched on.

There are key features of email interruptions that mean they are likely to represent a tempting proposition for Conscientious people. Interruptions, unlike distractions and cognitive interference (e.g., daydreaming), afford a task or action in their own right (Brixey *et al*., 2007; Fallows, 2002; Wickens & Hollands, 2000), which may be further compounded by organizational norms to respond quickly to email (Barley, Meyerson, & Grodal, 2011; Brown *et al*., 2014; Nurmi, 2011). Conscientious people, when faced with a potential task that needs checking to plan and prioritize work, are likely to be faced with a dilemma. On the one hand, the perseverance and self‐discipline of Conscientious people means that in order to maintain focus on their current task, resistance is needed to prevent the interruption demanding attention, and thus hindering achievement of current work goals and tasks. However, given their elevated levels of work investment and dutifulness, resisting the temptation to check email interruptions may demand especially strong self‐control from highly Conscientious people because incoming mail may indeed contain the requirements of a potential new task that could take higher priority (Frese & Zapf, 1994; Brown *et al*., 2014). This line of reasoning is consistent with TAT, by which the contextual demands of the email interruption alongside current task demands represent a complex and interacting set of organizational, social, and task‐related demands, which collectively activate individual Conscientiousness (Ent, Baumeister, & Tice, 2015; Fishbach *et al*., 2003; Hofmann, Baumeister, Förster, & Vohs, 2012). We propose, in sum, that whilst people ordinarily have a more consistent inclination to attend to the interruption promptly (i.e., the email is a temptation), in the presence of current task demands, those who are higher on the Conscientious dimension are rather more likely to resist the temptation in order to focus on the current task.

Research evidence supports this proposition and has demonstrated that people with higher levels of Conscientiousness can apply self‐control strategies more effectively, or for longer, in the face of temptations (Elfhag & Morey, 2008; Tangney *et al*., 2004). This appears to be due to Conscientious people being able to commit to achieving tasks that have already been planned for (Claessens, Van Eerde, Rutte, & Roe, 2010), being less able/willing to adapt actions to cope with changing task demands (LePine, Colquitt, & Erez, 2000) and being determined to focus on protecting their current task goals (Barrick, Mount, & Gupta, 2003). In particular, those able to resist engaging in immediate responding when alerted to the presence of an email interruption are also more likely to be persevering (stay on task) and apply premeditation (think through the consequences of action) – both of which are key facets of Conscientiousness (Whiteside & Lynam, 2001). In support of this, Fishbach *et al*.'s (2003) research found that individuals exerting high levels of self‐control ignored distracting events and avoided temptations that drew them away from another task. These findings inform our first hypothesis around the main effect of Conscientiousness on email interruption checking:

*H1*: People with higher levels of Conscientiousness take longer to check an email interruption, after receiving an alert at work.

The literature on self‐control shows that when experiencing strain or demands at work, it is more difficult to apply self‐control strategies, as deploying self‐control is draining (Fishbach *et al*., 2003). Interruptions are often classed as ‘demands’ (Schaufeli & Bakker, 2004), ‘hassles’ (Zohar, 1999), or obstacles to goal pursuit (Bailey *et al*., 2001). Empirical research reveals that those who have higher levels of Conscientiousness generally cope with environmental stressors better, and experience fewer health complaints and negative outcomes, often because they have better strategies for coping with demands (Lodi‐Smith *et al*., 2010; Luo & Roberts, 2015). Moreover, as we have earlier argued, elevated work investment, industriousness, and dutifulness associated with high Conscientiousness are likely to manifest in greater perseverance and commitment to tasks in the face of high demands and strain. COR further outlines how people who have more resources are better able to deal with environmental stressors, and subsequently experience less resource loss or threat (Hobfoll, 2001, 2011).

We propose that at work, in times of higher demand and increased strain, Conscientious people are therefore considered to execute better and more effective resource deployment strategies in goal pursuit, compared to their less Conscientious counterparts (Conner & Abraham, 2001; Lin *et al*., 2015; Penney, David, & Witt, 2011; Witt *et al*., 2002). Additionally, at times of high demand or increased strain, the application of self‐control strategies for highly Conscientious people will be particularly apparent because they are better able to draw on their resources when stressors increase (Halbesleben *et al*., 2009). This is typical for those who possess greater levels of key resources (Hobfoll, 2001). As such we predict that at times of high demand, people high on Conscientiousness will be substantially more effective than those low on Conscientiousness at resisting an email interruption:

*H2*: The relationship between Conscientiousness and time taken to check an email interruption after receiving an alert at work will be moderated by the perceived level of strain experienced at the point when the interruption alert was received, such that the relationship becomes more positive as strain increases.

Frei *et al*. (1999) found that people who limit their attention towards interrupting events tend to produce more work, but also experience more negative affect, because of the costs involved in this kind of self‐regulation. In particular, exercising self‐control to avoid an interruption increases cognitive load, as workers must engage memory processes to remember there is a new interrupting task to deal with, whilst trying to stay on track with a current work task (Gutzwiller, Wickens, & Clegg, 2014; Elfering *et al*., 2005), something that can be stressful (Einstein *et al*., 2003; Morgan *et al*., 2009). Such research demonstrates that applying a key resource strategy can deplete another, more volatile resource (such as AWB) in the pursuit of goals (work output). We have argued that this is likely to be augmented for those with higher levels of Conscientiousness, as they are more disposed towards attending to work tasks expeditiously, and so delaying dealing with a potentially competing task (the interruption) will likely be a more stressful experience for them (Hagger *et al*., 2010). Conscientious people feel compelled to persevere with current work tasks in the face of new demands (Whiteside & Lynam, 2001), even though their dutifulness may mean that they want to appraise new information in order to prioritize effectively (Judge, Simon, Hurst, & Kelley, 2014). On top of the normal cognitive demands associated with delaying dealing with an interruption, we reason that this quandary is likely to increase strain further for a Conscientious person. We propose that the longer it takes for the Conscientious person to check the competing work task (i.e., the length of the delay before attending to the interruption), the more likely this is to negatively impact their AWB (which is depleted in a trade‐off with the activation of Conscientiousness to exert self‐control behaviour strategies). This informs our next hypothesis:

*H3*: Taking longer to check an email interruption is (a) associated with reduced levels of AWB afterwards and (b) this relationship will be moderated by Conscientiousness, such that when those with higher levels of Conscientiousness take longer to check an email interruption at work, they will experience a greater reduction of AWB afterwards.

### Goal achievement and conscientiousness

It is apparent that applying a resource in the pursuit of one goal may necessarily hinder the achievement of another. For example, the pursuit of promotion at work may involve applying resources for time and effort, which can inhibit satisfaction of a person's other goals – such as the desire to be a present parent (ten Brummelhuis & Bakker, 2012; Grandey & Cropanzano, 1999; Greenhaus & Beutell, 1985; Halbesleben *et al*., 2009; Ilies *et al*., 2007). In multigoal environments we are faced with an ongoing process of prioritization (Louro, Pieters, & Zeelenberg, 2007), with resources being allocated to the most salient goals during any particular performance episode (Beal, Weiss, Barros, & MacDermid, 2005). Moreover, if two goals conflict, then they will compete for resources and inhibit each other (Kruglanski *et al*., 2002).

Research conducted in the context of COR has demonstrated that one resource can service multiple goals (multifinality: Kruglanski *et al*., 2002; Kruglanski, Chernikova, Babush, Dugas, & Schumpe, 2015) and multiple resources can service one goal (equifinality: Kruglanski *et al*., 2002, 2015). Discussion about resources that may service one goal but to the detriment of another goal has been rather limited (with some notable exceptions, e.g., Halbesleben *et al*., 2009; Louro *et al*., 2007). This is not because studies of goal conflict do not exist (see, e.g., Carver & Scheier, 1990; Kruglanski *et al*., 2002), rather, within COR, the association of resource value with the goals they serve has been missing in resource definitions until recently (Halbesleben *et al*., 2014). Understanding how resources are used to service different goals in a trade‐off arrangement further explicates our knowledge about resource deployment in COR.

In Hockey's cognitive‐energetical compensatory control model (1997, 2000, 2002), this trade‐off hypothesis is explained. Hockey reports that when self‐control is used to protect the current task goal, then this is usually at the expense of well‐being goals, as energy reserves are depleted and strain is experienced. Hockey (2002) also acknowledges that personality differences exist in terms of which goals are prioritized at such times: a problem‐focused person will adopt a task goal achievement protection strategy, paying for this with well‐being resources; an emotion‐focused person will adopt a strain resistant strategy, paying for this with task goal achievement resources. In the context of TAT, Barrick *et al*. (2013) suggest that different goals are more or less relevant to different manifestations of personality traits. If one is focused on working in the service of objectives (Achievement Striving) then one is more likely to be high on levels of Conscientiousness.

In this study then, we do not expect that *in general* those who take longer to check an email will report greater levels of task goal achievement or lower levels of well‐being goal achievement afterwards. However, we would expect, based on Hockey's trade‐off hypothesis, that those high in Conscientiousness who employ a task‐focused strategy (i.e., delay dealing with email interruptions to focus on current tasks), would report higher levels of task goal achievement, but simultaneously reduced well‐being goal attainment. Such an empirical observation would be consistent with the pattern of proposed resource use in the presence of email interruption (i.e., activation and expression of Conscientiousness as a key stable resource, and depletion of AWB as a volatile resource). This reasoning leads to our final two hypotheses:

*H4*: People with higher levels of Conscientiousness, who take longer to check an email interruption at work, will report greater levels of task goal achievement afterwards.

*H5*: People with higher levels of Conscientiousness, who take longer to check an email interruption at work, will report lower levels of well‐being goal achievement afterwards.

### The present study

In researching goal‐directed activity and affect variables, Ilies, Aw, and Lim (2016) and Ohly, Sonnentag, Niessen, and Zapf (2010) state the importance of establishing ecological validity in research designs. When undertaking experimental tasks, people's goals will not reflect the goals they prioritize in authentic work environments (Frese & Zapf, 1994). For example, participants are more likely to work flat out on a task to please an experimenter/gain course credit, etc. at the expense of well‐being goals; in the ‘real’ world, well‐being goals are given more priority (Hockey, 1997, 2000, 2002). Ecological Momentary Assessment (EMA: Brief & Weiss, 2002) and Experience Sampling Methods (ESM: Csikszentmihalyi & Larson, 1987) are used within studies concerned with capturing fluctuations in relationships between work‐relevant variables as embedded in people's normal work domain (Xanthopoulou, Bakker, & Ilies, 2012). EMA and ESM approaches, ‘…permit access to ongoing everyday behaviour in a relatively unobtrusive manner by gathering reports of events, experiences, and feelings close to when they happen’ (Conway & Briner, 2002, p. 289).

As such, to investigate whether Conscientious people are more resistant to email interruptions, and whether this impacts on subsequent AWB and perceptions of goal achievement, an EMA/ESM repeated‐measures methodology was adopted (Dimotakis, Scott, & Koopman, 2011) with working adults as they dealt with their normal incoming email in a field setting. At level one, time taken to check an email interruption (representing self‐control deployment) was linked with perceptions of AWB and goal achievement close to the time at which the experience occurred. At level two, Conscientiousness, as a personality characteristic, was measured as a direct predictor of self‐control deployment and in cross‐level interaction terms. This approach enables the examination of within‐person responses to interruptions as they occur in the workplace, and the between‐person variables that might influence these (Ohly *et al*., 2010; van Eerde, Holman, & Totterdell, 2005).

## Method

### Participants

Seventy four participants from four organizations were recruited for this study, via opportunity sampling by email or paper memo (via internal mail systems). The final number of participants included in the study was 52, owing to drop‐out rates, spoiled or insufficient data being returned. This is considered to be an equitable sample size to other repeated‐measures diary‐based studies conducted in the field (Dimotakis *et al*., 2011; Elfering *et al*., 2005; Miner, Glomb, & Hulin, 2005; Trougakos, Beal, Green, & Weiss, 2008). The incentives of entry into a small monetary prize draw and free, confidential personality feedback were offered. The 52 participants were knowledge workers drawn from a range of industry sectors, including manufacturing (13%), IT securities (8%), hygiene and logistics (8%), and academia (25% in academic positions; 46% in non‐academic positions). Two per cent were academic professorial or reader level, 12% were senior management or academic senior lecturer/researcher grade, 31% were middle or project management or academic lecturer/researcher grade, 21% were between administrative and management levels, or academic junior lecturers/researchers, and 33% were at administrative level or academic research assistant level. 54% of participants were female. The modal age range of participants was 21–30 (33%).

### Procedure

Participants were required to monitor their response to email interruptions over the course of half of a working day (lasting 4 hrs). Participants nominated a time period when they were expecting to be based at their normal work stations for the majority of the time. Paper and pencil information and record sheets were used (as per Elfering *et al*., 2005; Louro *et al*., 2007). At the start of the study period, participants were instructed to log on and download all of their email. They did not complete record forms for these email; only subsequent email, that interrupted the participant when based at their desk and engaged in another work activity, was to be assessed. This was to ensure that only email *as an interruption* was examined. During the course of the nominated study period, participants remained on‐line at all times and were interrupted by naturally occurring email alerts^1^ , received as part of their normal incoming email traffic. Participants were asked to respond to each email interruption as they usually would.

Participants completed an email record immediately after they had finished processing any email that had interrupted their work, and before returning to their interrupted task. The email record form had been previously trialled in two pilot studies and one other study to ensure sense, validity and expedience. A personality questionnaire was administered on‐line within 1‐week of the study period.

### Measures

#### Control measures^2^

As the demands of a task and the demands of the interruption can both provoke a more or less rapid response time (Gutzwiller *et al*., 2014; Wickens, Gutzwiller, & Santamaria, 2015), impact goal achievement (Altmann & Trafton, 2002), and well‐being (Bailey *et al*., 2001), parameters of the interrupted task and interrupting email were captured as control measures, according to how Lengthy, Difficult, Clear and Specific, and Effortful they were. Each parameter was rated on a 6‐point scale where 1 = ‘not at all’ and 6 = ‘very much’. Ratings were summed across the four parameters (‘Clear and Specific’ was reverse scored) to give a score for ‘Demanding Task’ and ‘Demanding Email’. Higher scores indicate higher demands for the interrupted task and interrupting email, respectively.

Further, the number of the email interruption just dealt with was recorded on the participants’ forms (e.g., ‘4’ indicates the fourth email interruption dealt with during the study period). This variable is labelled ‘Email Number’ and is included as a control variable as recent research suggests that the cumulative effect of interruptions can impact strategy choice and outcomes (Baethge, Rigotti, & Roe, 2015). This is important in the context of self‐control studies. If time taken to check an email reduces as Email Number increases, then this suggests direct self‐control resource depletion in line with COR (and previous studies of self‐control), an effect which needs to be controlled.

#### Email checking time

The length of time elapsing before the participant checked the email interruption following an alert (Wickens *et al*., 2015) was measured by asking participants, ‘how long did it take you to check the message after receiving the alert (estimate to the nearest second)?’ Subjective ratings were captured as there is no inherent, objective time stamp available in the email record to indicate the time that elapses between alert receipt and email ‘checking’. A high score indicates a slower ‘Checking Time’, as more time will have elapsed between receiving the email alert and checking the email content, indicative of exercising greater self‐control. Checking Time was skewed and leptokurtic, and so was transformed using logarithmic transformation and standardization to improve the distribution.

#### Momentary AWB

The ten‐item version of Daniels’ (2000) five‐factor AWB (D‐FAW) questionnaire was used to measure momentary AWB at two points. These items have been validated for use in work contexts and experience‐sampling diary research (Harris, Daniels, & Briner, 2003). Four items (Anxious; At Ease, reversed; Annoyed; Calm, reversed) designed to measure Negative Affect (NA) were used in this study. For each email record, participants were asked to rate on a six‐point scale the extent to which ‘you feel this way right now, that is, at the present moment’ (where 1 = ‘not at all’ and 6 = ‘very much so’) for each item. Cronbach's alpha was calculated on 376 cases (from this study) as 0.98. This variable is referred to as ‘Momentary NA’. Note that a high NA score indicates lower AWB, pertaining to Hypothesis 3.

In addition, for each email record, participants were asked to rate on a six‐point scale ‘how you felt right before being interrupted by the email alert’ (where 1 = ‘not at all’ and 6 = ‘very much so’) for each D‐FAW NA item (as outlined above). Cronbach's alpha was calculated on 376 cases (from this study) as 0.78. This variable is referred to as ‘Before NA’^3^ . A high score indicates higher negative affect at the point when the email interruption was received (indicates the degree of strain the participant was experiencing – relevant to Hypothesis 2). A high score on ‘Momentary NA’ or ‘Before NA’ represents a strain experience, and is indicative of low AWB.

#### Self‐reported goal achievement

Participants were asked to consider the degree to which their strategy for dealing with the email interruption helped or hindered them in ‘achieving your current task's goal’ or ‘in achieving a sense of personal well‐being’. Variables are labelled as ‘Perceived Task Goal Achievement’ and ‘Perceived Well‐being Goal Achievement’, respectively, and were rated on a three‐point scale, where 1 = goal hindered, 2 = goal neither helped nor hindered, and 3 = goal helped.

#### Personality

The Hogan Personality Inventory (HPI: Hogan & Hogan, 1997) is a 206‐item questionnaire, based on the Five‐Factor Model of personality. It uses a forced choice true‐false response format and is fully standardized and validated for a working population (Hogan & Hogan, 1997; Salgado, 2003). There are seven primary scales. Participants must complete the questionnaire in its entirety, but for this study only the scale measuring Conscientiousness (HPI Prudence: 31 items) was of interest. The scale was standardized to the z‐scale before analysis. Cronbach's α reliability statistics are provided by the UK test publisher – Psychological Consultancy Ltd – and is .87 for the HPI Prudence scale. Participants completed the questionnaire on‐line using specialist administration and scoring software within five working days of the nominated study period.

### Analyses

Hierarchical Linear Modelling (HLM: Snijders & Bosker, 2004) using random coefficients (Kreft & deLeeuw, 2004) was employed to analyse our data. There were two levels to the data. At level‐1 repeated‐measures data from each email record form was used (*n* = 376). This was nested within the individual participants (*N* = 52) at level‐2. All level‐1 variables were person‐mean centred to limit confounding effects from between‐person variance (Bono, Glomb, Shen, Kim, & Koch, 2013; Trougakos *et al*., 2008) including common‐method (self‐report) variance (Dimotakis *et al*., 2011).

In all of the analyses, MLWiN version 3 was used (Rasbash, Browne, Healy, Cameron, & Charlton, 2016). Having established that a two‐level model was a better fit for the data than the null model, a forward‐stepping procedure was adopted to prevent over‐inflation of results (Hofmann *et al*., 2012; Nezlek, 2003). In Step 1, control variables were entered. In Step 2, predictor variables were added. If the value for the predictor variables was significant at *p *<* *.05, the variable was retained; if not, it was removed and Step 2 was run again (final model reported), unless the variable was needed for the interaction term in Step 3. In Step 3, interaction terms were tested. In all steps, variables were entered as fixed coefficients (random intercepts only), to avoid reduction in power that can occur when too many parameters are included in a model (Kreft & deLeeuw, 2004). Improvement in fit at each step is based on improvements in chi squared from the final model represented in the previous step.

## Results

During the nominated study period, between 2 and 20 interrupting emails were received per participant, with an average of 7.23 and a median of 4. Table 1 provides descriptive statistics (means and standard deviations) for each of the study variables, along with intercorrelations at level‐1.

**Table 1:** Descriptive statistics and intercorrelations between explanatory variables

	Variable	*N*	Mean	SD	1	2	3	4	5	6	7	8
1	Email Number	376	5.15	3.73								
2	Demanding Task	353	12.47	4.98	.07							
3	Demanding Email	358	8.40	4.12	.02	.13*						
4	Momentary NA	342	2.45	0.92	.05	.20**	.26**					
5	Before NA	350	2.42	0.89	.14**	.23**	.16**	.89**				
6	Checking Time	376	0.00	1.00	.19**	−.07	−.00	.05	.04			
7	Perceived Task Goal Achievement	375	1.83	0.71	.09	−.26**	−.11*	−.06	−.01	.20**		
8	Perceived Well‐being Goal Achievement	374	2.22	0.63	−.00	−.06	−.07	−.13*	.01	−.06	.22**	
9	Conscientiousness	336	0.00	1.00	.05	.06	.07	−.25**	−.19**	.22**	.07	.05

Notes

(1) Checking Time has been subject to logarithmic transformation and standardization, hence its mean of ‘0’ and S.D. of ‘1’. When calculations are conducted on the raw data, the mean time taken to check is 275.86 s (approximately 4 and a half minutes), with a standard deviation of 644.92 s; this is heavily skewed however, and the median time to check is: 0 seconds (i.e., immediate checking on alert); (2) ‘Conscientiousness’ is Level‐2 data that has been standardized, but this has been entered into this analysis at Level‐1, meaning that the same score is repeated for each candidate's Level‐1 data correlations; (3) Two‐tailed Pearson's product moment correlations are significant at *p <* *.05; **p <* *.01.

Four models were run. Table 2 shows results from the first two models. Model 1 tested Hypotheses 1 and 2, and Model 2 tested Hypothesis 3. Table 3 shows results from the third and fourth models, which test hypotheses 4 and 5 respectively.

**Table 2:** Models 1 and 2

	Step 1: Entering controls	Step 2: Entering predictors	Step 3: Entering moderators
Model 1: Predictors of Checking Time			
Intercept	−0.05 (.10)	0.04 (.11)	0.04 (.11)
Control variables			
Email Number	0.02 (.01)*	0.02 (.02)	0.01 (.02)
Demanding Email	0.01 (.01)	0.02 (.01)*	0.01 (.01)
Demanding Task	−0.02 (.01)*	−0.01 (.01)	−0.01 (.01)
Fixed effects			
Before NA		−0.01 (.10)	0.01 (.10)
Conscientiousness		0.23 (.10)**	0.23 (.10)**
Interaction effects			
Conscientiousness* Before NA			0.32 (.11)**
Model			
Level 1 variance	0.60 (.05)**	0.65 (.06)**	0.63 (.06)**
Level 2 variance	0.40 (.10)**	0.36 (.10)**	0.36 (.10)**
2*Log likelihood	893.11 (*N* = 349)	787.05 (*N* = 301)	778.30 (*N* = 301)
Improvement in fit (χ^2^)	59.50** (3 df) From null model	106.06** (2 df) From Step 1 model	8.75** (1 df) From Step 2 model
Model 2: Predictors of Momentary NA after processing an email interruption			
Intercept	2.43 (.11)**	2.45 (.11)**	2.45 (.11)**
Control variables			
Email Number	−0.01 (.01)	−0.01 (.01)	−0.01 (.01)
Demanding Email	0.05 (.01)**	0.04 (.01)**	0.04 (.01)**
Demanding Task	0.01 (.01)	0.01 (.01)	0.01 (.01)
Fixed effects			
Checking Time		0.08 (.04)*	0.07 (.04)*
Conscientiousness		−0.15 (.11)	−0.15 (.11)
Interaction effects			
Checking Time*Conscientiousness			0.11 (.04)**
Model			
Level 1 variance	0.30 (.03)**	0.28 (.03)**	0.27 (.02)**
Level 2 variance	0.48 (.11)**	0.46 (.11)**	0.47 (.11)**
2*Log Likelihood	642.00 (*N* = 327)	564.46 (*N* = 294)	555.57 (*N* = 294)
Improvement in fit (χ^2^)	52.54** (3 df) From null model	77.54** (2 df) From Step 1 model	8.89** (1 df) From Step 2 model

Note

Two‐tailed significance: *p <* *.05; **p <* *.01. Standard errors are in parentheses (all level 1 predictors are person‐mean centred).

**Table 3:** Models 3 and 4

	Step 1: Entering controls	Step 2: Entering predictors	Step 3: Entering moderators
Model 3: Predictors of Perceived Task Goal Achievement			
Intercept	1.77 (.07)**	1.79 (.07)**	1.79 (.07)**
Control variables			
Email Number	−0.00 (.01)	−0.01 (.01)	−0.01 (.01)
Demanding Email	−0.01 (.01)	−0.01 (.01)	−0.01 (.01)
Demanding Task	−0.02 (.01)*	−0.02 (.01)*	−0.02 (.01)*
Fixed effects			
Checking Time		0.02 (.04)	0.08 (.04)
Conscientiousness		0.07 (.07)	0.07 (.07)
Interaction effects			
Conscientiousness* Checking Time			−0.01 (.04)
Model			
Level 1 variance	0.31 (.03)**	0.32 (.03)**	0.32 (.03)**
Level 2 variance	0.18 (.05)**	0.17 (.05)**	0.17 (.05)**
2*Log likelihood	660.61 (*N* = 349)	604.45 (*N* = 316)	604.35 (*N* = 316)
Improvement in fit (χ^2^)	47.77 ** (3 df) From null mode l	56.16** (2 df) From Step 1 model	0.10 (1 df) From Step 2 model
Model 4: Predictors of Perceived Well‐being Goal Achievement			
Intercept	2.22 (.01)**	2.26 (.01)**	2.26 (.01)**
Control variables			
Email Number	−0.01 (.01)	−0.01 (.01)	−0.01 (.01)
Demanding Email	−0.01 (.01)	−0.01 (.01)	−0.01 (.01)
Demanding Task	0.00 (.01)	−0.00 (.01)	−0.00 (.01)
Fixed effects			
Checking Time		−0.12 (.04)**	−0.11 (.04)**
Conscientiousness		0.05 (.05)	0.05 (.05)
Interaction effects			
Checking Time*Conscientiousness			−0.08 (.04)*
Model			
Level 1 variance	0.27 (.02)**	0.26 (.02)**	0.25 (.02)**
Level 2 variance	0.12 (.03)**	0.09 (.03)**	0.09 (.03)**
2*Log likelihood	599.06 (*N* = 348)	518.40 (*N* = 315)	512.68 (*N* = 315)
Improvement in fit (χ^2^)	34.44** (3 df) From null model	80.66** (2 df) From Step 1 model	5.72* (1 df) From Step 2 model

Note

Two‐tailed significance: *p < .05; **p < .01. Standard errors are in parentheses (all level 1 predictors are person‐mean centred).

**Figure 1:**
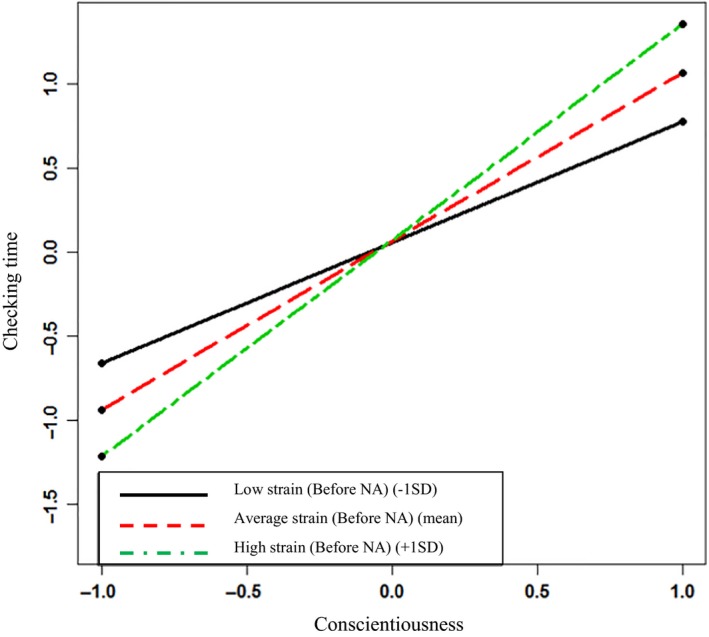
Strain (negative affect at the point of email interruption) as a moderator of the relationship between Conscientiousness and Checking Time. [Colour figure can be viewed at wileyonlinelibrary.com]

In Step 1 of Model 1, Email Number (γ_ij_ = .02; *p = *.02) and Demanding Task (γ_ij_ = −.02; *p = *.02) were initially significantly and positively related to time taken to check an email interruption. Demanding Email was not significant (γ_ij_ = .01; *p = *.16). In Step 2, only Demanding Email was significant (γ_ij_ = .02; *p = *.02), but none of the controls were significant in Step 3. In Step 2 of the model, Before NA was not a significant predictor (γ_ij_ = −.01; *p = *.46), but Conscientiousness has a significant and positive relationship with time taken to check an email interruption (γ_j_ = .23; *p = *.01). This supports Hypotheses 1, indicating that the higher the level of Conscientiousness, the longer it takes to respond to an email interruption after alert (the trait‐relevant self‐control strategy). Although Before NA was not significant at Step 2, it was retained in the model as it was then used in the cross‐level interaction term with Conscientiousness at Step 3. Here, the cross‐level interaction term is significant (γ_ij_ = .32; *p *=* *.002). To establish if this interaction effect was in the hypothesized direction a simple slopes analysis for a 2‐way multilevel model (with cross‐level interactions) was run (Preacher, Curran, & Bauer, 2006). Figure 1 presents the plot of the interaction, and the slopes analysis shows that Before NA significantly moderates the relationship between Conscientiousness and Checking Time when Conscientiousness is low (1 *SD* below the mean: γ = −.31, SE = .14, *z *=* *2.19*, p *=* *.03), and high (1 *SD* above the mean: γ = .33, SE = .14, *z *=* *2.33*, p *=* *.02), although not at a mean level (γ = .01, SE = .10, *z *=* *0.10*, p *=* *.92). This interaction effect is in the hypothesized direction; effectively, the higher one's level of Conscientiousness the slower one is to check an email interruption, and this is especially so if one feels more anxious and annoyed when the interruption arrives. Those with lower levels of Conscientiousness take less time to check an email interruption when they feel anxious and annoyed when the interruption arrives. This supports Hypothesis 2.

**Figure 2:**
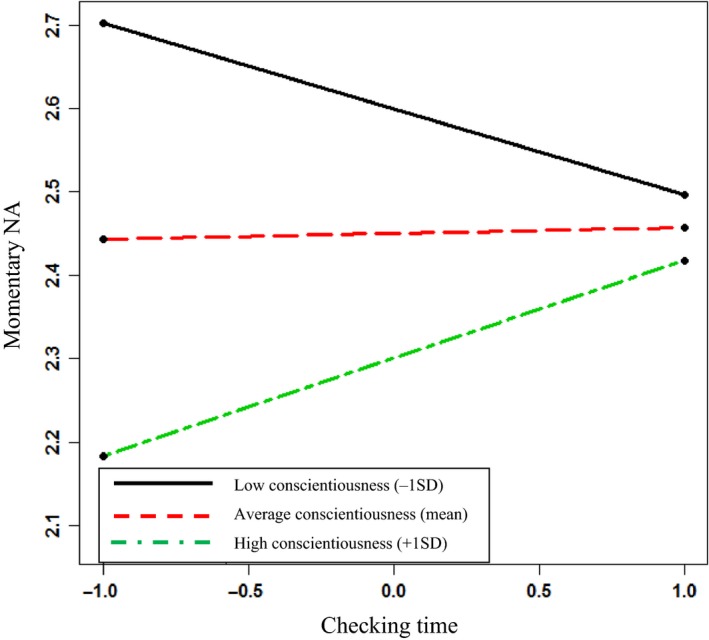
Conscientiousness as a moderator of the relationship between email Checking Time and Momentary NA (afterwards). [Colour figure can be viewed at wileyonlinelibrary.com]

Model 2 examines whether the deployment of self‐control (indicating activation of the Conscientiousness resource) is linked to reduced AWB resources afterwards (operationalized here as higher Momentary NA). In Step 1 of Model 2, Email Number (γ_ij_ = −.01; *p = *.16) and Demanding Task (γ_ij_ = .01; *p = *.16) were not significant, but Demanding Email has a significant, positive relationship with Momentary NA after dealing with the email interruption (γ_ij_ = .05; *p < *.001), and this retained significance across the subsequent steps. In Step 2 of Model 2, Checking Time is a significant predictor of Momentary NA after dealing with an email interruption (γ_ij_ = .08; *p = *.02), although Conscientiousness is not significant (γ_j_ = −.15; *p = *.09), providing support for Hypothesis 3(a). Although Conscientiousness is not significant at Step 2, it was retained in the model as it was then used in the cross‐level interaction term with Checking Time at Step 3. Here, the cross‐level interaction term is significant (γ_ij_ = .11; *p *=* *.003). To establish whether this interaction effect was in the hypothesized direction, a simple slopes analysis was run as before. Figure 2 presents the plot of the interaction, and the slopes analysis shows that Checking Time is positively and significantly related to Momentary NA when Conscientiousness is high (1 *SD* above the mean: γ = .12, SE = .06, *z *=* *2.07*, p *=* *.04). However, the simple slopes are not significant when Conscientiousness is low (1 *SD* above the mean: γ = −.10, SE = .06, *z *=* *−1.82*, p *=* *.07), or at a mean level (γ = .01, SE = .04, *z *=* *0.18*, p *=* *.86). Looking at Figure 2, the slope for highly Conscientious people (at 1 *SD* above the mean) shows that as Checking Time increases, so too does their Negative Affect. However, NA is still at a relatively low level, this interaction supports Hypothesis 3(b), in that when a more Conscientious person delays checking their email after alert (applies self‐control) he/she reports lower levels of AWB.

Model 3 explores Hypothesis 4 and whether Conscientious people who take longer to check email may be deploying self‐control resources despite the negative impact on well‐being (see Model 2), because it helps them to achieve their current task goal more effectively. However, neither the main effects nor the interaction term were significant predictors of Perceived Task Goal Achievement. Only the control variable ‘Demanding Task’ predicted Perceived Task Goal Achievement in the negative direction (γ_ij_ = −.02; *p = *.02). As seen, in the final step of Model 3 the cross‐level interaction term of Conscientiousness and Checking Time was not significant (γ_ij_ = −.01; *p *=* *.40). As such, Hypothesis 4 is rejected.

**Figure 3:**
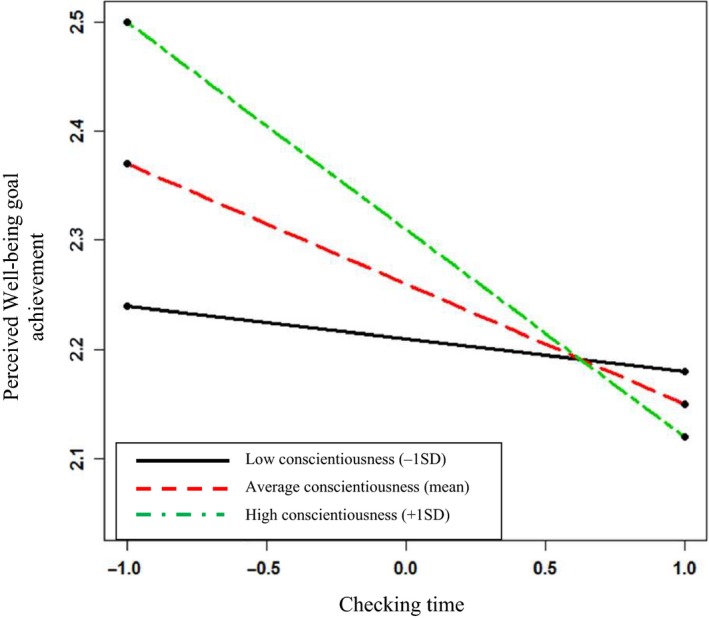
Conscientiousness as a moderator of the relationship between email Checking Time and Perceived Well‐being Goal Achievement. [Colour figure can be viewed at wileyonlinelibrary.com]

In Model 4 there were significant findings, offering support to Hypothesis 5. None of the control variables had a significant effect on Perceived Well‐being Goal Achievement. However, in Step 2, taking less time to check an email after being interrupted significantly predicted Perceived Well‐being Goal Achievement (γ_ij_ = −.12; *p < *.001). Checking Time retains significance as a main effect in Step 3 (γ_ij_ = −.11; *p *=* *.003), and as part of the interaction term with Conscientiousness. Here, Checking Time significantly and negatively predicts Perceived Well‐being Goal Achievement and this relationship is strengthened by degree of Conscientiousness (γ_ij_ = −.08; *p = *.02). Figure 3 presents the plot of the interaction, and the simple slopes analysis (as before) shows that the negative relationship between Checking Time and Perceived Well‐being Goal Achievement is moderated by Conscientiousness when Conscientiousness is high (1 *SD* above the mean: γ = .19, SE = .04, *z *=* *4.25*, p < *.001) and at a mean level (γ = .11, SE = .03, *z *=* *−3.48*, p < *.001). However, the simple slopes are not significant when Conscientiousness is low (1 *SD* below the mean: γ = −.03, SE = .04, *z *=* *0.67*, p *=* *.50). Looking at Figure 3, it can be seen that for highly Conscientious people (1 *SD* above the mean) Perceived Well‐being Goal Achievement is higher when they take less time to check the email. Perceived Well‐being Goal Achievement reduces for highly Conscientious people as Checking Time increases (i.e., as self‐control is employed), whereas there is little impact here for those with low Conscientiousness. Because this demonstrates that taking longer to check an email has a negative impact on the perceived achievement for well‐being goals for highly Conscientious people, Hypothesis 5 is supported by these findings.

## Discussion

The findings of the present study reveal insights into the interplay of stable and volatile resources in the context of work tasks and interruptions. Our findings make new theoretical contributions to the literature on COR. In particular, our methodology enabled us to observe and analyse the dynamics of resource deployment and depletion in response to commonplace daily work demands: email interruptions when working on a task. Our results permit us to advance understanding of the effects of stable resource deployment (i.e., activation and application of Conscientiousness), on volatile resource depletion (i.e., reduction in AWB).

People with higher levels of Conscientiousness were more resistant to checking email interruptions at work, supporting Hypothesis 1 that Conscientious people are more likely to adopt self‐control behavioural strategies when faced with off‐task demands. Highly Conscientious people were especially resistant to checking email interruptions when experiencing strain (low AWB) at the point of interruption, compared with those with lower levels of Conscientiousness, who showed less self‐control in resisting the allure of an email interruption at such times. These findings offer support to the research indicating that Conscientious people are more likely to apply self‐control behaviours and stay on task for longer, when faced with temptations, especially at times of strain (Elfhag & Morey, 2008; Hagger *et al*., 2010; Tangney *et al*., 2004; Whiteside & Lynam, 2001), supporting Hypothesis 2. This also supports COR in its proposition that possessing a key resource is beneficial in resisting demands and applying effective resource deployment (Halbesleben *et al*., 2009; Hobfoll, 2011), and indicates the importance of selecting people to work within any particular context, who possess the key resources necessary to deal with job demands.

However, in applying a self‐control strategy, highly Conscientious people were also more susceptible to experiencing lowered AWB afterwards. Whilst delaying checking an email interruption increased negative affect for all (i.e., reduces AWB: supporting Hypothesis 3a), this was especially the case for Conscientious people (Figure 2), supporting Hypothesis 3b. This suggests that in deploying aspects of a key resource (taken from the stable end of the volatile‐stable resource dimension), other, more volatile resources (from the opposite end of the spectrum) may be depleted; in this case AWB was spent. This finding makes a significant contribution to COR theory by examining the impact that the application of stable resources has on more volatile resources. For our study participants, volatile resources were expended when stable resources were deployed.

Moreover, in Model 1 Step 1, Checking Time increased in line with the number of email interruptions a person had received. This suggests that the tendency to resist the email interruption was strengthened over repeated exposure, not weakened. This could offer greater support for TAT's position that using a trait‐relevant strategy strengthens rather than weakens that resource. More research is needed now to test this finding using other key resources with trait‐relevant deployment strategies.

Reflecting previous work on the trade‐off in goal‐directed behaviour (ten Brummelhuis & Bakker, 2012; Grandey & Cropanzano, 1999; Greenhaus & Beutell, 1985; Halbesleben *et al*., 2009; Ilies *et al*., 2007), we proposed (hypotheses 4 and 5) that Conscientious people, when faced with an email interruption, adopt an email‐resistance strategy to protect current task achievement goals, even though this may be at the expense of their well‐being goals (Hockey, 1997, 2000, 2002). However, this proposition was only partly realized. We found that a delaying strategy did not predict greater perceived task goal achievement for Conscientious people (failing to support Hypothesis 4), although it did predict reduced perceived well‐being goal achievement for Conscientious people (supporting Hypothesis 5). Combined with the fact that AWB suffers after deploying self‐control, it seems clear that this strategy does have a negative impact on well‐being as both a resource and a goal for Conscientious people. This pattern of findings has implications for the conceptual development of COR theory in terms of the nature of traits as key stable resources, AWB as a more volatile resource, and the behavioural mechanisms that potentially regulate between the two. We discuss this now in the following sections.

### Conscientiousness as a key resource

Our results present an initial paradox for the conceptualization of Conscientiousness as a key resource that is deployed in the service of goal‐directed activity. Given that Conscientious people value task goal achievement (Witt *et al*., 2002; Witt, Andrews, & Carlson, 2004; Halbesleben *et al*., 2009), why do Conscientious people use strategies for avoiding or resisting email interruptions, if (as observed in our data) perceived task goal achievement is unaffected? Indeed, engaging Conscientiousness to resist an email interruption (1) did not affect perceived achievement of either a well‐being or task goal (van den Broeck, Vansteenkiste, De Witte, & Lens, 2008; Halbesleben *et al*., 2014), but neither did it (2) result in subsequent reduced resistance and shorter checking times (as seen in Model 1, step 1).

Conscientiousness does not therefore appear to operate in ways that are conventionally observed for ‘resources’. That is, it is not depleted, and its application is not necessarily associated with people's perceptions of goal achievement. Is it therefore justified to consider Conscientiousness as a resource? We propose that this question can be potentially addressed by applying the logic of TAT. In TAT, traits are activated in complex ways in response to specific task, social and organizational contextual cues. Conscientiousness is a broad bandwidth personality trait, activated in different ways by different contextual triggers. Email interruptions, we have argued, are especially salient for highly Conscientious people, and in one respect activate behavioural strategies to stay on task. In another respect these prompt self‐control strategies to resist quick email checking that would follow from activation of dutifulness. We may assume that other features of focal tasks, and organizational context (e.g., normative expectations) are also activating Conscientiousness in different ways.

That we did not observe an effect on Perceived Task Goal Achievement may therefore reflect the more complex nature of the deployment of Conscientiousness as a resource at work. At the level of people's perceptions, although we asked participants to specifically comment on task goal achievement with regard to the current task that was interrupted, it is plausible that Conscientious people were unable to consider that they had successfully attained a current task goal, if other work goals (e.g., those afforded by the email interruption) were being compromised in the process. Alternatively, at the behavioural level, it may be that people do not perceive narrow behavioural manifestations of, for example, current task‐focus (e.g., resisting email interruptions), as directly or uniquely serving task goal achievement, but the broader strategy of attending to and differentially prioritizing one's multitude of work tasks nevertheless might. In sum, we argue for a potential positioning of traits as a special form of key resource, deployed in specific and complex ways across multiple work activities. The challenge for researchers moving forwards will be to unpick this complexity in order to understand how stable traits and their associated behavioural strategies are deployed to service goals in the context of the task and organization, and how this impacts volatile resources. In practice, providing guidance on the ‘best’, or contextually most appropriate, strategy to apply to meet organizational or task goals, may help Conscientious people to feel less conflicted about what to do when faced with two options that might equally satisfy different trait‐relevant goals (counter‐finality: Kruglanski *et al*., 2015). Choosing a strategy that has been endorsed by the organization (e.g., ‘check all email on receipt of alert’, or, ‘turn off your email alerts when focused on another task’) may have less of a depleting impact on volatile resources such as AWB, especially because Conscientious people are likely to be more rule‐abiding. This needs to be tested but indicates a potential practical benefit in organizations providing explicit policies (or even potentially an implicit culture) when email strategy best practice is unclear.

### Well‐being as a volatile resource

It is notable in our research that resisting an email interruption directly predicted negative affect afterwards and hindered the achievement of well‐being goals. Although resistance times did not decline within‐participants, maintaining self‐control as a strategy *was* draining, much more so for people high in Conscientiousness. This key personality dimension therefore appears to indirectly determine the rate at which AWB as a resource is depleted in work activities for different people.

Work context undoubtedly plays a role in these relationships, as proposed in TAT. If Conscientious people experience a degradation in well‐being, through deploying self‐control behaviour in response to work cues, then it is sensible for steps to be taken in work design that reduce unnecessary resistance to natural behavioural inclination. For example, in our study context, an email interruption is, we argue, a temptation for people high on Conscientiousness, and resistance is deployed to stay on task. This activation and resistance mechanism could be avoided if email systems were switched off – attending to email tasks as planned, rather than ‘interrupting’ activities (McFarlane, 2002; Mark, Iqbal, Czerwinski, Johns, & Sano, 2016). To protect AWB and avoid unnecessary strain, the clear implication is that task and job design should take account of individual differences in quite specific ways.

### Behavioural mechanisms and the regulation of resource deployment and depletion

The nature of behaviour expressed through the activation of personality traits, is that it is habitual and automatic (Tett & Burnett, 2003), and in effect, traits represent consistent patterns of response to situational cues. Automatic responding is thought to preserve resources, and is the regulatory process applied in familiar circumstances (Frese & Zapf, 1994). Fishbach *et al*. (2003) report that strategies to avoid temptations can become automated over time. Habituating strategies towards a stressor will reduce its impact (Zellars, Hochwarter, Lanivich, Perrewé, & Ferris, 2011), but in becoming automated, such strategies can lose their purpose, becoming somewhat rigidly applied, without thought, and without attending to potential goal achievement (Baumeister *et al*., 2007). In dealing with email interruptions, a Conscientious person may have devised a self‐control strategy that was originally intended to protect task goals, but which, from repeated use, is now automatically applied when the interruption event appears, and has become purposefully redundant.

In a situation where people are dealing with a very common event, such as an email interruption (Speier *et al*., 2003), resistance to checking that email may represent a habitual, rather than conscious, response. Nevertheless, reduction in AWB was observed after the email interruption had been dealt with, consistent with the exertion of self‐control (Baumeister *et al*., 2007). This pattern of findings raises the prospect that the behavioural mechanisms, such as self‐control, that regulate between deployment of key stable resources on one hand, and volatile resources on the other, may be automatic and determined by work context rather than individual volitional choices.

Examining how resource deployment becomes automated, and the impact this has on both resource loss and goal achievement will be an intriguing future research strand. This may be especially warranted because as demands on workers are increasing exponentially in our modern working world (Derks, ten Brummelhuis, Zecic, & Bakker, 2014; Gorgievski *et al*., 2011) shortcuts may be applied with the purpose of increasing efficiency, but with the effect of cueing behavioural patterns for specific workers that sets them on a pathway of AWB depletion, and if left unchecked, exhaustion. In sum, this dynamic trade‐off of resources, whilst observed at an event‐focused level in our study, is a potential pathway for understanding the roots and processes of ‘over‐work’.

### Limitations

We used a repeated‐measures EMA/ESM approach, as this allowed us to capture data about email interruptions in real time and in the context of people's real goals and priorities. This methodology provides a highly ecologically‐valid field study setting for our research, and greatly adds to the applied significance of our findings. Despite these notable strengths, there are some limitations that are pertinent to keep in mind in respect of all non‐laboratory, diary‐based research (see Conway & Briner, 2002; and the 2005 special edition on diary studies in the Journal of Occupational and Organizational Psychology). Firstly, we were mindful that ‘recall’ of the email event, and the self‐reporting of checking time, might be distorted if the duration of time from the receipt of the email to the completion of the email diary record (completed when the email interruption had finished being dealt with) was lengthy. Accurate recall is a drawback of self‐report event‐sampling techniques, and our study is subject to this limitation. However, the impact is likely to have been no greater than in other self‐report diary studies; indeed many apply retrospective ratings with much greater periods between events and ratings (e.g., Trougakos *et al*., 2008). Secondly, and more generally, there is a possibility that participation in the study may alter the natural behavioural tendencies of participants (Orne, 1962; Symon, 2000). This again is common to diary‐based methods and although great care was taken to explain to participants the need to behave as they normally would at work, the possibility cannot be totally discounted. Our reasonably long sampling period (4 hrs) mitigates this to some degree.

Self‐report methodology also has limitations in terms of common‐method variance. However, our level‐1 data (AWB and email Checking Time) was collected at a different time point to level‐2 data (personality), consistent with suggestions for limiting error (Podsakoff, MacKenzie, Lee & Podsakoff, MacKenzie, Lee, & Podsakoff, 2003). The absence of large intercorrelations between the variables also mitigates concerns that method bias inflated ratings (Grandey & Cropanzano, 1999).

Relatedly, we acknowledge a potential confound of personality on post‐hoc ratings of experience. For example, Conscientious people typically have high performance standards and may be self‐critical in respect of their appraisal of work events (Halbesleben *et al*., 2009; Hofmann *et al*., 2012). As such, they may self‐report that actions taken to respond to interruptions reduced well‐being and did not result in effective goal achievement, even if in reality their outputs were successful. In future, it would be judicious to take an objective measure of task goal achievement.

Our study used an opportunity sampling approach and as such participants were not matched by job type, normal email load or current task. We asked participants to rate the demands of the task they were working on when interrupted, and included this as a control measure in the study. We also used ‘email number’ as a control variable – to account for the notion that someone working on their twentieth email interruption of the day may take a quite different approach to someone working on their third. However, future research in samples of people working in more homogeneous work environments (e.g. in high‐interruption environments, or high‐concentration job roles) could be of interest to further unpick how job role and environment may impact people's behaviour response to those interruptions.

Finally, we recognize that in respect of self‐control behaviour, we have followed a precedent approach that positions resistance to a temptation as evidence that a self‐control strategy had been adopted (Baumeister, Bratslavsky, Muraven, & Tice, 1998; Hofmann *et al*., 2012; Muraven & Baumeister, 2000; Vohs & Schmeichel, 2003). However, it would be valuable for future studies building on ours, to consider explicitly measuring behavioural expression of traits such as Conscientiousness in strategies like self‐control, to develop a richer picture of the behaviours deployed as key resource traits are activated.

### Conclusion and final comments

In this EMA/ESM field study of how people deploy resources to deal with email interruptions, we identified that Conscientiousness, as a key resource and stable personality characteristic, was activated via self‐control strategies to resist the interruption for longer. Applying Conscientiousness in this way had a depleting effect on another resource – that of AWB. Such a finding expounds understanding of resource deployment within COR, as it illustrates how using a key resource does not necessarily result in its loss, but the loss of other, more volatile, and expendable resources at the other end of the stability spectrum – such as AWB. Our findings further revealed that whilst well‐being goals were perceived to be hindered when Conscientious people executed a self‐control strategy to resist interruptions, task goals were neither perceived to be helped nor hindered. And yet, the deployment of the self‐control strategy appeared to strengthen over the duration of the study.

In examining why Conscientious people utilize self‐control strategies in response to email interruption, without there being a discernible perceived benefit to either task or well‐being goal achievement, we utilized both COR and TAT theories to provide potential explanations. Firstly, we posit that the presence of an email interruption may prompt an activation‐resistance mechanism for Conscientious people who may simultaneously want to exercise self‐discipline and persevere with their current task whilst conflictingly wanting to respond to the email task out of a sense of duty and responsiveness. Thus, the worker is unable to conceive that the strategy used (self‐control) either helps or hinders the achievement of task goals. We also used the most current conceptualization of resources in COR – as factors that are valuable in the context of goal pursuit – to ask whether a resource is still a resource if its execution in the pursuit of goals has become automated to the point whereby goals are no longer perceived to be fulfilled. This question would benefit from attention in future studies. It also implies that if people are applying automated strategies for dealing with their work email, which have little benefit for their well‐being or their tasks, then *all* workers may benefit from being periodically reminded to review the approach taken to dealing with work demands – to examine whether resource deployment strategies really are as purposeful, functional and efficient as they should be.

## Footnotes

1 40% received a floating message preview box on‐screen, 60% received an envelope icon alert, 46% received an audible alert.

2 Please note that we attempted to abide by the principles for use of Control Variables, set out by Bernerth and Aguinis (2016). They recommend that control variables should only be included in a study if they can be theoretically justified, are central to our hypothesis testing in terms of focal variables, already have an empirically established relationship with focal variables, and affect results to the extent that they offer an alternative explanation. As such, and in order to heighten power and parsimony in model fit, we did not include the other personality characteristics from the HPI as control variables in any of our models. The reader may like to know however, that we did run all of our models with personality characteristics as controls, just to test the impact they had on results. In no model were our findings challenged by the inclusion of the other FFM characteristics. Only one factor (Neuroticism – HPI Adjustment) showed a significant relationship with an outcome, and this was for Momentary NA in Model 2. However, this did not negate the effects of our predictor variables and interaction term, and so was not included in our final model, as we had no interest in this study in testing or confirming the relationship between Neuroticism and Affect.

3 Retrospective ratings of affect are commonly used in well‐being measurement (where people rate how they felt over the course of the past day or week, for example). Because ‘Before NA’ is rated by participants at the same time as ‘Momentary NA’ we are mindful that ratings may take the form of a comparison, whereby the scores given may reflect the participant implicitly comparing ‘how do I feel now compared to then’. This may give a relative, rather than absolute, value of affect.
